# Adaptive free-space optical communications through turbulence using self-healing Bessel beams

**DOI:** 10.1038/srep43233

**Published:** 2017-02-23

**Authors:** Shuhui Li, Jian Wang

**Affiliations:** 1 Wuhan National Laboratory for Optoelectronics, School of Optical and Electronic Information, Huazhong University of Science and Technology, Wuhan 430074, Hubei, China

## Abstract

We present a scheme to realize obstruction- and turbulence-tolerant free-space orbital angular momentum (OAM) multiplexing link by using self-healing Bessel beams accompanied by adaptive compensation techniques. Compensation of multiple 16-ary quadrature amplitude modulation (16-QAM) data carrying Bessel beams through emulated atmospheric turbulence and obstructions is demonstrated. The obtained experimental results indicate that the compensation scheme can effectively reduce the inter-channel crosstalk, improve the bit-error rate (BER) performance, and recuperate the nondiffracting property of Bessel beams. The proposed scheme might be used in future high-capacity OAM links which are affected by atmospheric turbulence and obstructions.

Over the past decades, free-space optical communication (FSO) which provides an easy means of high-bit-rate communications has attracted more and more interest as an adjunct or alternative to radio relay link line of sight (LOS) communications^1^. Favored by bandwidth, spectrum and security issues, it is expected that FSO links can be widely used in the future military and civilian networks, such as communications between spacecraft, temporary network installation for events or other purpose as disaster recovery, up-and-down links between space platforms and ground platforms, deep-space probes, Last-Mile access, and security applications^2^. Very recently, using orthogonal spatial modes in FSO systems has shown the potential to tremendously increase system capacity and spectral efficiency by multiplexing data-carrying beams in the same spectral band^3^,^4^,^5^,^6^,^7^. Several different types of orthogonal modal basis sets have been presented as potential candidates for FSO systems. In particular, one such set is orbital angular momentum (OAM)^8^.

An OAM-carrying light beam is featured by a helical phase front of exp (*ilφ*), where *l* is the topological charge number and φ refers to the azimuth angle^9^. Typically, a well-known representation of helically phased beam is the so-called Laguerre–Gaussian (LG) laser beam^10^. Most attention in the literature regarding OAM-carrying beams has been dedicated to LG beams. However, the LG beam is not the only example of OAM-carrying beams; Bessel beams, Mathieu beams, and Ince–Gaussian beams can also carry orbital angular momentum^11^. Although all these modal sets are orthogonal and complete, there exit differences between LG beams with other OAM modal sets which may lead to different potential applications. In particular, Bessel beams having the property of propagation-invariant or diffraction-free have generated considerable interest^12^,^13^,^14^. Very recently, by using the self-healing mechanism (reconstruct itself after encountering an obstruction) and orthogonality of Bessel beams, it has shown the possibility to establish the obstruction-free or obstruction-tolerant FSO OAM communication links^15^,^16^.

In FSO links, a critical challenge is the atmospheric turbulence which will cause random fluctuations in amplitude and phase of the laser beams. These fluctuations can lead to an increase in the link error probability, limiting the performance of communications systems^17^. Moreover, for OAM multiplexing systems, the turbulence may also cause crosstalk between multiple channels, resulting in significant performance degradation of signal^18^. Although OAM-carrying Bessel beam can reconstruct itself after encountering obstruction, the strong turbulence will also impair the nondiffracting property causing phase and intensity distortions. In order to mitigate the influence of turbulence, adaptive optics based compensation techniques have been proposed for OAM-carrying LG beam multiplexing systems, showing favorable performance^19^,^20^,^21^. However, so far there has been limited report in studying the performance of Bessel beam multiplexing link through atmospheric turbulence and compensation block.

In this paper, we propose an obstruction- and turbulence-tolerant FSO OAM multiplexing link by using self-healing Bessel beams assisted by adaptive compensation techniques. A spatial light modulator (SLM) loaded with Kolmogorov phase mask added by an obstruction phase mask is used to simultaneously emulate atmospheric turbulence and obstruction in the laboratory environment. By measuring the phase distortions of a probe Gaussian beam through a wavefront sensor (WFS), correction masks are created and loaded to another SLM to compensate the distortions of multiplexed Bessel beams. Using this scheme, we experimentally demonstrate compensation of multiple Bessel beams through emulated atmospheric turbulence and obstruction. The compensation effects on inter-channel crosstalk and bit-error rate (BER) performance of a two Bessel beams multiplexing system each carrying a 10-Gbaud (40-Gbit/s) 16-ary quadrature amplitude modulation (16-QAM) data are studied.

## Results

### Concept and principle

**Figure 1:**
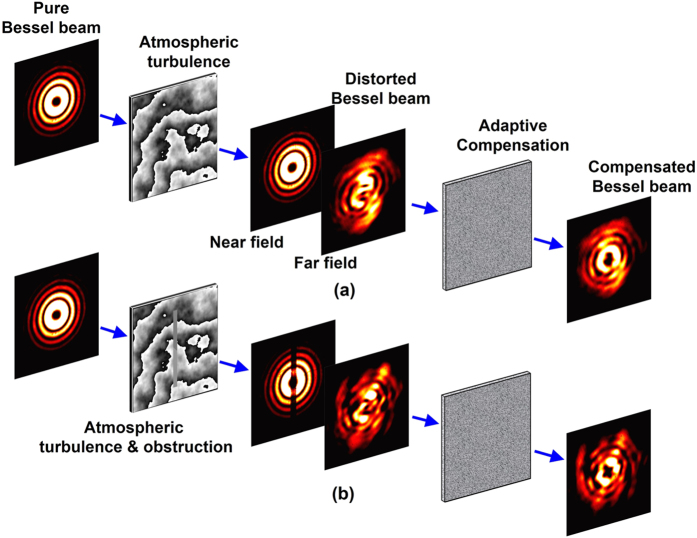
(**a**) Propagation of Bessel beam through turbulence assisted by adaptive compensation (**b**) Propagation of Bessel beam through turbulence and obstruction assisted by adaptive compensation.

Figure 1 shows the block diagram of turbulence compensation for distorted Bessel beam propagation links. Two cases are considered here: (i) compensation for a distorted link with only atmospheric turbulence (Fig. 1(a)); (ii) compensation for a distorted link with both atmospheric turbulence and obstruction (Fig. 1(b)). In the first case, only phase distortions caused by atmospheric turbulence are studied. A phase screen is used to emulate the atmospheric turbulence in the laboratory environment. The field just after the phase screen, i.e. the near field, has almost the same intensity profile as input Bessel beam but distorted phases. After diffraction transmission, the far field becomes distorted, losing the characteristics of the Bessel beam. In order to recover the Bessel beam, an adaptive compensation unit is introduced to correct the phase distortions. While for the second case, both phase distortions caused by atmospheric turbulence and obstructions are considered. Due to the self-healing property of Bessel beam, it could be reconstructed itself after encountering a partial obstruction. However, the atmospheric turbulence will destroy the nondiffracting property, resulting in distorted beam profiles. Fortunately, after turbulence compensation, the self-healing property can still be recuperated.

### Experimental setup

**Figure 2:**
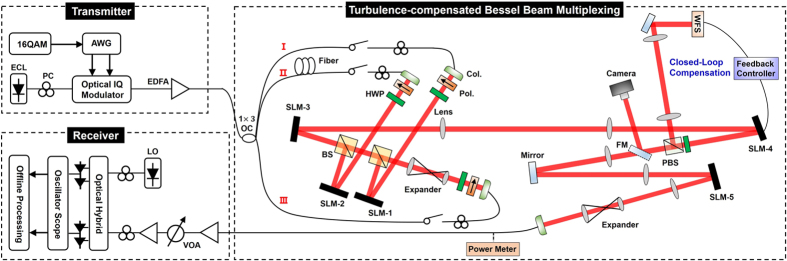
Experiment setup. ECL: External cavity laser, PC: polarization controller, AWG: arbitrary waveform generator, EDFA: erbium-doped fiber amplifier, OC: optical coupler, SLM: spatial light modulator, Col.: collimator, Pol.: polarizer, HWP: half-wave plate, BS: non-polarizing beam splitter, PBS: polarizing beam splitter, FM: flip mirror, VOA: variable optical attenuator, LO: local oscillator.

The experiment setup for demonstrating compensation of distorted multiplexed Bessel beams is illustrated in Fig. 2. The transmitter block is used to generate 10-Gbaud (40-Gbit/s) 16-QAM signal at a wavelength of 1550 nm. The signal is then split into three paths and fed into three collimators, generating three collimated Gaussian beams (the beam radius of path I and II is about 2 mm). One beam (path I) is launched to SLM-1 loaded with a hologram phase mask to create one Bessel beam with topological charge *l* = 3. The second beam (path II) is injected to SLM-2 and converted to another Bessel beam with topological charge *l* = 5. Bessel beams are generated by holographic axicon masks^22^. The propagation-invariant distance of generated Bessel beam is about 0.74 m. And the distance between SLM-1 and SLM-3 is about 0.36 m. It is noteworthy that path II is turned on only when studying the impact of crosstalk on the BER of multiplexed Bessel beams. The third Gaussian beam (path III) after expansion is used as the probe beam for phase distortion sensing and required correction-mask retrieval. Then the beams are sent to a turbulence emulator (SLM-3) loaded with pseudo-random phase masks that follow the Kolmogorov spectrum statistics^23^. The distorted beams exiting from SLM-3 are imaged onto the wavefront corrector (SLM-4) loaded with correction masks for turbulence compensation. In order to obtain the correction mask, the probe Gaussian beam firstly passes through the link. A small portion of the probe beam reflected by SLM-4 is imaged onto a Shack–Hartmann WFS (HASO from Imagine Optic Inc.) by using a 4*f* lens system for phase distortion detection. Then a feedback controller dynamically feeds SLM-4 with the appropriate correction masks according to difference between target phase and measured phase. The correction mask obtained from the probe Gaussian beam is then used to compensate the multiplexed Bessel beams. A probe beam is adopted as the reference of phase distortion sensing due to the fact that it is hard to directly measure the wavefront distortion of an OAM beam having doughnut shape (i.e. null intensity at the beam center) with Shack-Harman WFS. Note that probe beam and multiplexed Bessel beams could be separated by using different polarizations or wavelengths^20^,^21^. After wavefront correction, the multiplexed Bessel beams are then sent to SLM-5 loaded with changeable phase mask for demultiplexing followed by coherent detection.

**Figure 3:**
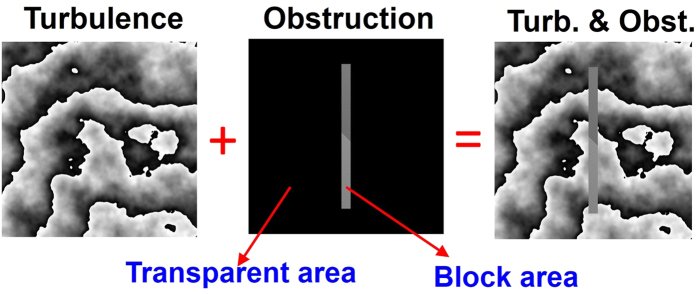
Method of creating the phase mask used to simultaneously emulate atmospheric turbulence and obstruction.

We use pseudo-random phase masks that follow the Kolmogorov spectrum statistics to emulate atmospheric turbulence characterized by the Fried coherence length *r*_0_^24^. An example of Kolmogorov turbulence phase mask with *r*_0_ = 1 mm is shown in Fig. 3. The emulated propagation distance through atmosphere is 1 km. Moreover, to simultaneously emulate atmospheric turbulence and obstruction with single phase mask, an obstruction phase mask is added to the turbulence phase mask, as depicted in Fig. 3. The obstruction phase mask has transparent area and block area. The block area is actually blazed grating with small period which can diffract the input beam to a specific direction. However, the transparent area will not affect the direction of the input beam. When the grating period is small enough, limited by the size of optical elements, such beam will be obstructed. Moreover, for a more realistic situation, obstruction can be emulated by a real opaque object, while the SLM is only used to emulate atmospheric turbulence.

## Experimental results

**Figure 4:**
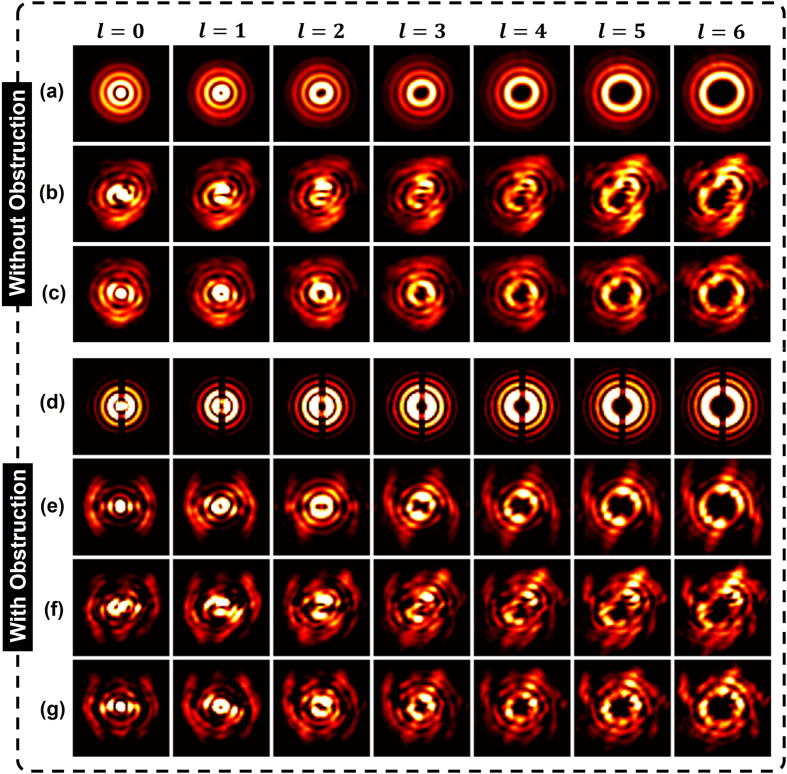
(**a**) Measured intensity profiles of input Bessel beams without turbulence and obstruction. (**b**,**c**) Measured far-field intensity profiles for various Bessel beams before and after compensation with only turbulence. (**d**,**f**) Measured near-field and far-field intensity profiles for various Bessel beams after the turbulence-obstruction phase mask. (**e**) Measured far-field intensity profiles for various Bessel beams with only obstruction. (**g**) Measured far-field intensity profiles for various Bessel beams after compensation with turbulence and obstruction.

We first transmit the probe Gaussian beam through a random turbulence realization with *r*_0_ = 1 mm and use the adaptive close-loop to generate the corresponding correction mask. Then the correction mask is used to compensate the wavefront distortions of Bessel beams from charge *l* = 0 to charge *l* = 6. For the case with only turbulence, the measured field profiles of various Bessel beams with and without compensation are shown in Fig. 4(a–c). From the figures, one can see that the distorted Bessel beams are efficiently compensated. For the case with strip obstruction and turbulence, the measured near and far field profiles are displayed in Fig. 4(d–g). The impact of the obstruction can be seen from the near field profiles just after the turbulence-obstruction phase mask (Fig. 4(d)). Bessel beams can reconstruct themselves after traveling a distance, as shown in Fig. 4(e). However, from Fig. 4(f), one can find that the self-healing property will be distorted by atmospheric turbulence. By using the compensation, the self-healing property can be recuperated, as displayed in Fig. 4(g).

**Figure 5:**
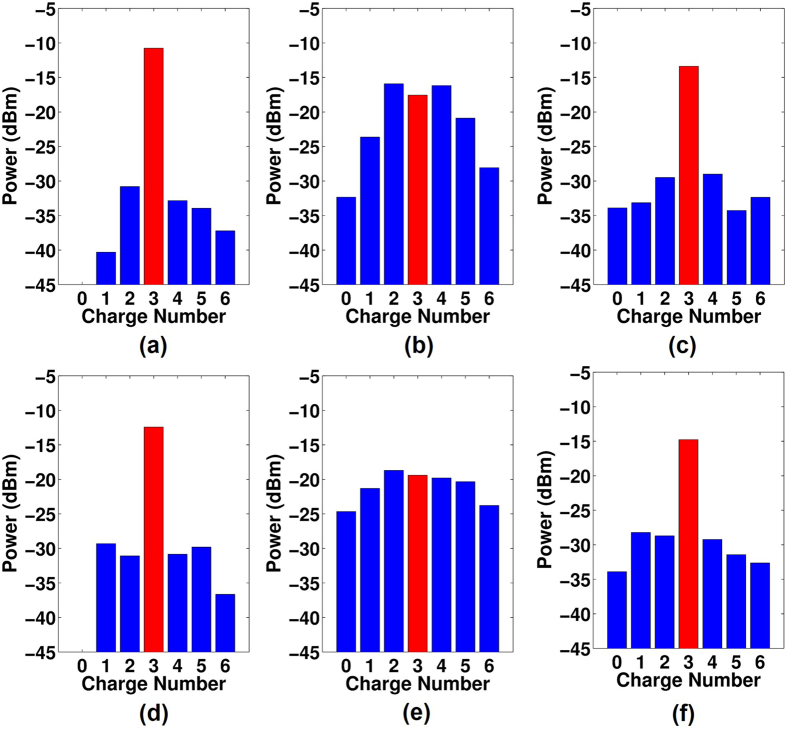
Measured power distributions of various channels when only Bessel *l* = 3 is transmitted. (**a**) Without turbulence and obstruction. (**b**) With a random turbulence realization. (**d**) With only strip obstruction. (**e**) With strip obstruction and a random turbulence realization. (**c**,**f**) The measured compensated results corresponding to (**b**) and (**e**), respectively.

Figure 5 shows the measured power distributions over different channels when only Bessel beam *l* = 3 is transmitted. Both cases without obstruction (Fig. 5(a–c)) and with strip obstruction (Fig. 5(d–f)), under a random turbulence realization, with and without compensation are measured. The wavefront distortion caused by turbulence may lead to power leakage from a specific Bessel beam to the neighboring channels, causing inter-channel crosstalk, as shown in Fig. 5(b,e). Figure 5(d) shows the measured power distributions of various channels with strip obstruction. Owing to the self-healing property of Bessel beams, the inter-channel crosstalk caused by the strip obstruction is not obvious, but it may induce some power loss. After compensation, the measured results are depicted in Fig. 5(c,f). From the power distributions, one can find that without compensation, the power is spread among neighboring channels, whereas with compensation, the received power is better confined to the Bessel channel *l* = 3. Moreover, the self-healing property can be recuperated, as shown in Fig. 5(f).

**Figure 6:**
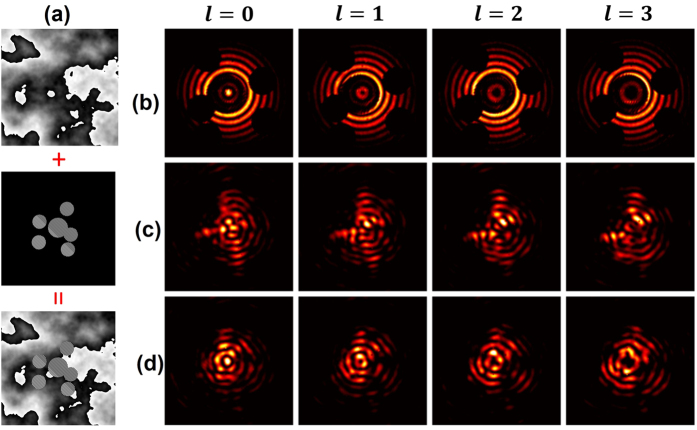
(**a**) Phase mask for simultaneously emulating random circular obstruction and atmosphere turbulence. (**b**) Measured near-field intensity profiles for various Bessel beams. Measured far-field intensity profiles for various Bessel beams with (**d**) and without (**c**) compensation.

Moreover, we measured the near and far filed intensity profiles of Bessel beams passing through a more complicated random emulated obstruction which consists of a number of randomly distributed circular block area as shown in Fig. 6(a). From Fig. 6(b–d), it can be seen that the self-healing property can also be recuperated through adaptive compensation.

**Figure 7:**
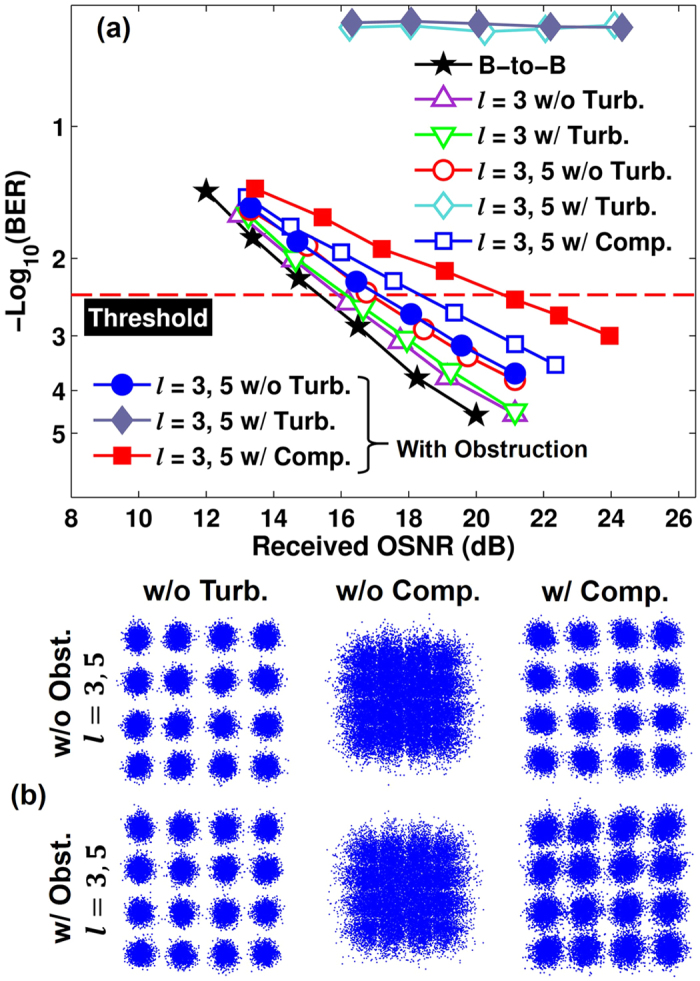
(**a**,**b**) Measured BERs and constellations for Bessel channel *l* = 3 when Bessel beams *l* = 3 and 5 transmit through the link, with and without obstruction, under a random turbulence realization, with and without compensation.

Finally, we measure the BER performance of 10-Gbaud (40-Gbit/s) 16-QAM signals over multiplexed Bessel channels with a random turbulence realization. Path I is used to transmit data-carrying Bessel beam *l* = 3. Meanwhile, path II is also turned on to transmit data-carrying Bessel beam *l* = 5 to investigate the crosstalk effects. The measured BERs and constellations for Bessel channel *l* = 3, with and without compensation are shown in Fig. 7. Both cases with and without obstruction are measured. When only channel *l* = 3 passes through the system, the turbulence just cause received power fluctuation but no crosstalk. Therefore, the power penalty is very small. When both channel *l* = 3 and *l* = 5 are turned on, due to the strong crosstalk caused by turbulence, the BER values do not decease with the increase of optical signal-to-noise ratio (OSNR), and cannot be below the forward error correction (FEC) limit of 3.8e-3. After compensation, the BERs could achieve the FEC limit with small OSNR penalties compared to the cases without turbulence. For the case without obstruction, after compensation the measured crosstalk from channel *l* = 5 to *l* = 3 is reduced from −3.3 dB to −21.2 dB. For the case with obstruction, the measured crosstalk is reduced from −2.1 dB to −17.5 dB.

## Discussion

We have experimentally demonstrated an OAM-carrying Bessel beam multiplexing link for adaptive FSO communications through turbulence. By using the self-healing property, it is possible to establish obstruction-free FSO OAM communication links. However, strong atmospheric turbulence may distort the phase and intensity of Bessel beam causing inter-channel crosstalk and impairing the nondiffracting property. To realize obstruction- and turbulence-tolerant OAM FSO links, adaptive compensation approach has been introduced to the Bessel multiplexing system. Using a SLM loaded with Kolmogorov phase mask added by an obstruction phase mask, we simultaneously emulate atmospheric turbulence and obstruction in the laboratory environment. The atmospheric turbulence compensation scheme is similar to that scheme for LG beams, using a beacon beam to measure wavefront aberrations. Due to the phase distortion and obstruction are at the same plane, therefore, the compensation scheme can be also used for Bessel beams with obstruction. However, for a more complicated situation, obstruction may also cause phase distortions, and some improved methods should be used to compensate the turbulence. The compensation effects on inter-channel crosstalk and BER performance of a two Bessel beams multiplexing system each carrying a 10-Gbaud (40-Gbit/s) 16-QAM data are demonstrated. The obtained experimental results indicate that the compensation scheme can effectively suppress inter-channel crosstalk, improve the BER performance, and recuperate the nondiffracting property of Bessel beams. The proposed scheme might see potential applications in future high-capacity OAM links which are affected by atmospheric turbulence and obstructions.

## Methods

### Generation of Bessel beams

A Bessel beam can be generated by a phase mask with the transmission function given as


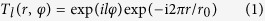


where *l* is the order of a Bessel beam, and *r*_0_ is an adjustable constant parameter. For such a Bessel beam the nondiffracting distance can be estimated as^22^


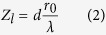


where *d* is the radius of input beam.

### Turbulence Emulation

We employ an phase-only spatial light modulator (SLM) with a resolution of 1920 × 1080 pixel to emulate the atmospheric turbulence. The refractive-index power spectral density of the turbulence phase mask is computed by^25^


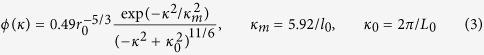


where *L*_0_ is called the outer scale, *l*_0_ is called the inner scale, and *r*_0_ is the Fried coherence length.

## Additional Information

**How to cite this article**: Li, S. and Wang, J. Adaptive free-space optical communications through turbulence using self-healing Bessel beams. *Sci. Rep.* **7**, 43233; doi: 10.1038/srep43233 (2017).

**Publisher's note:** Springer Nature remains neutral with regard to jurisdictional claims in published maps and institutional affiliations.

## References

[b1] Henniger H. & Wilfert O. An introduction to free-space optical communications. Radioengineering 19, 203–212 (2010).

[b2] Chan V. W. Free-space optical communications. J. Lightwave Technol. 24, 4750–4762 (2006).

[b3] Yan Y. . High-capacity millimetre-wave communications with orbital angular momentum multiplexing. Nat. Commun. 5, 4876 (2014). 25224763 10.1038/ncomms5876 PMC4175588

[b4] Wang J. . Terabit free-space data transmission employing orbital angular momentum multiplexing. Nature Photon. 6, 488–496 (2012).

[b5] Wang J. Advances in communications using optical vortices. Photon. Res. 4(5), B14–B28 (2016).

[b6] Milione G. . 4 × 20 Gbit/s mode division multiplexing over free space using vector modes and a q-plate mode (de) multiplexer. Opt. Lett. 40, 1980–1983 (2015). 25927763 10.1364/OL.40.001980

[b7] Zhao Y. & Wang J. High-base vector beam encoding/decoding for visible-light communications. Opt. Lett. 40, 4843–4846 (2015). 26512464 10.1364/OL.40.004843

[b8] Willner A. . Optical communications using orbital angular momentum beams. Adv. Opt. Photonics 7, 66–106 (2015).

[b9] Allen L., Beijersbergen M. W., Spreeuw R. & Woerdman J. Orbital angular momentum of light and the transformation of Laguerre-Gaussian laser modes. Phys. Rev. A 45, 8185 (1992). 9906912 10.1103/physreva.45.8185

[b10] Padgett M., Courtial J. & Allen L. Light’s orbital angular momentum. Phys. Today 57, 35–40 (2004).

[b11] Allen L. & Padgett M. The orbital angular momentum of light: An introduction. Twisted Photons: Applications of Light with Orbital Angular Momentum, First Edition, 1–12 (2011).

[b12] Durnin J., Miceli J. Jr & Eberly J. Diffraction-free beams. Phys. Rev. Lett. 58, 1499 (1987). 10034453 10.1103/PhysRevLett.58.1499

[b13] Garces-Chavez V., McGloin D., Melville H., Sibbett W. & Dholakia K. Simultaneous micromanipulation in multiple planes using a self-reconstructing light beam. Nature 419, 145–147 (2002). 12226659 10.1038/nature01007

[b14] Gatto A., Tacca M., Martelli P., Boffi P. & Martinelli M. Free-space orbital angular momentum division multiplexing with Bessel-Gauss beams. J. Opt. 13, 064018 (2011).

[b15] Ahmed N. . Experimental demonstration of obstruction-tolerant free-space transmission of two 50-Gbaud QPSK data channels using Bessel beams carrying orbital angular momentum. in *The European Conference on Optical Communication* (*ECOC*). paper, We.3.6.2 (2014).

[b16] Du J. & Wang J. High-dimensional structured light coding/decoding for free-space optical communications free of obstructions. Opt. Lett. 40, 4827–4830 (2015). 26512460 10.1364/OL.40.004827

[b17] Zhu X. & Kahn J. M. Free-space optical communication through atmospheric turbulence channels. IEEE Trans. Commun. 50, 1293–1300 (2002).

[b18] Ren Y. . Atmospheric turbulence effects on the performance of a free space optical link employing orbital angular momentum multiplexing. Opt. Lett. 38, 4062–4065 (2013). 24321923 10.1364/OL.38.004062

[b19] Zhao S., Leach J., Gong L., Ding J. & Zheng B. Aberration corrections for free-space optical communications in atmosphere turbulence using orbital angular momentum states. Opt. Express 20, 452–461 (2012). 22274368 10.1364/OE.20.000452

[b20] Ren Y. . Adaptive-optics-based simultaneous pre-and post-turbulence compensation of multiple orbital-angular-momentum beams in a bidirectional free-space optical link. Optica 1, 376–382 (2014).

[b21] Ren Y. . Turbulence compensation of an orbital angular momentum and polarization-multiplexed link using a data-carrying beacon on a separate wavelength. Opt. Lett. 40, 2249–2252 (2015). 26393711 10.1364/OL.40.002249

[b22] Paterson C. & Smith R. Higher-order Bessel waves produced by axicon-type computer-generated holograms. Opt. Commun. 124, 121–130 (1996).

[b23] Lane R., Glindemann A. & Dainty J. Simulation of a Kolmogorov phase screen. Waves Random Media 2, 209–224 (1992).

[b24] Fried D. L. Statistics of a geometric representation of wavefront distortion. J. Opt. Soc. Am. A 55, 1427–1431 (1965).

[b25] Andrews L. C. & Phillips R. L. Laser beam propagation through random media. Vol. 52 (SPIE press Bellingham, WA, 2005).

